# The New Carotenoid Pigment Moraxanthin Is Associated with Toxic Microalgae

**DOI:** 10.3390/md9020242

**Published:** 2011-02-10

**Authors:** Olga Mangoni, Concetta Imperatore, Carmelo R. Tomas, Valeria Costantino, Vincenzo Saggiomo, Alfonso Mangoni

**Affiliations:** 1 Dipartimento delle Scienze Biologiche, Università di Napoli Federico II, Via Mezzocannone 8, 80134 Napoli, Italy; Email: olga.mangoni@unina.it; 2 Dipartimento di Chimica delle Sostanze Naturali, Università di Napoli Federico II, via D. Montesano 49, 80131 Napoli, Italy; Email: cimperat@unina.it (C.I.); valeria.costantino@unina.it (V.C.); 3 Center for Marine Science, University of North Carolina at Wilmington, 5600 Marvin K. Moss Lane, Wilmington, NC 28409, USA; Email: tomasc@uncw.edu; 4 Stazione Zoologica "A. Dohrn", Villa Comunale I, 80121 Napoli, Italy; Email: saggiomo@szn.it

**Keywords:** *Chattonella cf. verruculosa*, Raphidophyceae, toxic algae, carotenoids, moraxanthin

## Abstract

The new pigment “moraxanthin” was found in natural samples from a fish mortality site in the Inland Bays of Delaware, USA. Pure cultures of the species, tentatively named *Chattonella cf. verruculosa*, and natural samples contained this pigment as a dominant carotenoid. The pigment, obtained from a 10 L culture of *C. cf. verruculosa*, was isolated and harvested by HPLC and its structure determined from MS and 1D- and 2D-NMR. The data identified this pigment as a new acylated form of vaucheriaxanthin called moraxanthin after the berry like algal cell. Its presence in pure cultures and in natural bloom samples indicates that moraxanthin is specific to *C. cf. verruculosa*  and can be used as a marker of its presence when HPLC is used to analyze natural blooms samples.

## 1. Introduction

Phytoplankton, unicellular photosynthetic microorganisms, are ubiquitous in all aquatic environments. As primary producers, they are responsible for nearly half of the global primary production of organic carbon [1]. Photosynthesis, the process whereby energy is absorbed by pigments in algae and transformed into chemical energy, relies on the presence of energy trapping pigments. The main pigments, chlorophylls, carotenoids and phycobilins, absorb Photosynthetically Available Radiation (PAR) from 400-700 nm wavelengths [2]. However, pigments may also serve several functions including metabolic regulation, light harvesting (antenna pigments), electron donation or acceptance (in reaction centers), and photoprotection. The combination of different pigments and functions result in maximum efficiency and economy [3,4,5,6]. The kind of pigments produced and their relative proportions characterize the different phytoplankton groups. 

In recent years, high performance liquid chromatography (HPLC) has been used to estimate phytoplankton composition by identifying photosynthetic pigments. Some pigments found exclusively in particular algal classes or genera may serve as useful taxonomic markers [7,8,9,10,11,12,13]. Such indicator pigments are termed ‘finger prints’. Pigment analyses offer a valuable technique in oceanography for mapping phytoplankton populations and monitoring their abundance and composition [14,15,16,17].

Phytoplankton blooms occur naturally in coastal waters particularly during spring and summer seasons. However, a small number of microalgae are harmful, and although each individual is small, they may occur in huge numbers known as blooms [18,19,20,21]. Among the estimated phytoplankton species, about 7% (300 species) are known to produce red tides and of those, only 2% are actually harmful or toxic [22]. In marine and brackish water environments, most toxic species belong in the Dinophyceae, but also the Diatomophyceae, Haptophyceae, Raphidophyceae, and Cyanophyceae comprise toxic species [23,24,25,26,27,28]. The algal toxins may cause damage to other flora and fauna directly or they may accumulate through the food web in e.g., shellfish or finfish, thereby causing harm to their predators including humans [29,30,31,32,33,34]. Harmful algal blooms (HABs) are an ever more frequent phenomenon expanding in coastal regions on a world scale [35,36,37,38]. These have received much attention from researchers and local regulatory authorities due to their impact on the ecosystem and human health, influencing local economic issues [39]. 

Monitoring of coastal waters for harmful species is costly and labor-intensive and the possibility to recognize a potentially harmful algal species by means of chemical or biochemical analyses significantly reduces the time and costs of such monitoring. The one caveat is that the analysis, pigment or biochemical, involves a species specific marker for the HAB species in question [40]. Pigment signatures in the study of HABs have been very limited, particularly in monitoring programs [33,41,42].

During the summer period of 2000, ten fish mortality events occurred from unidentified causes in the Inland Bays of Delaware, USA. During the final fish-killing event of 28 August, 2000, over two million menhaden (*Brevoortia tyrranus*) perished when a bloom of an unidentified microalgal flagellate was observed [36]. This flagellate, accompanied by the presence of a potent neurotoxin, was tentatively called *Chattonella* cf. *verruculosa* since it resembled a fish killing species found in Japan thought to be of the class Raphidophyceae. Since none of the previously described Raphidophyceae completely agreed with the molecular features (18S rDNA; 16S rDNA) [43,44], further studies are underway to define its taxonomic position.

**Figure 1:**
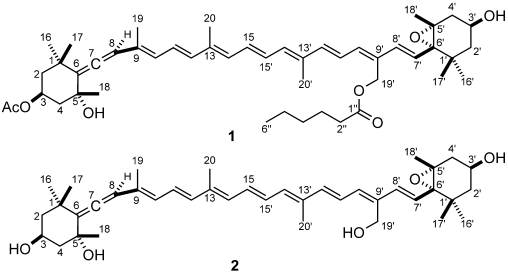
Structures of moraxanthin (**1**) and vaucheriaxanthin (**2**).

This work describes the isolation and structural elucidation of a new pigment (**1**) found in *C. cf. verruculosa* cultures and in natural samples where this species was dominant, which has been called moraxanthin after the berry like algal cell (Figure 1). Moraxanthin, which is a new acylated form of vaucheriaxanthin (**2**), is unique to *C. cf. verruculosa*, indicating that it can be used as a marker of its presence when HPLC analyses of natural blooms are performed.

## 2. Results and Discussion

**Figure 2:**
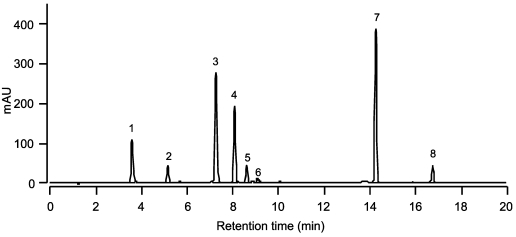
HPLC absorbance chromatogram of the extract from cultured *C. cf. verruculosa*.

**Figure 3:**
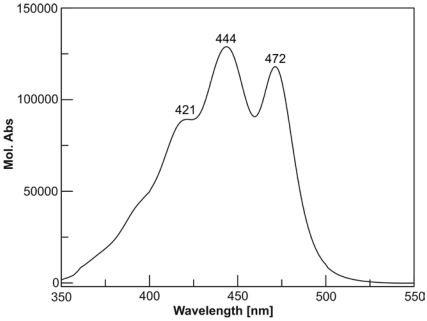
UV spectrum of moraxanthin **1** (EtOH).

The chromatogram of the pigments of the *C. cf. verruculosa* culture showed a major peak (Figure 2, peak 4), whose retention time (Table 1) and UV spectrum (Table 1 and Figure 3) did not fit those of any known pigments, although the UV spectrum clearly showed the pigment to be a carotenoid.

**Table 1:** Total pigments found in *C. cf. verruculosa* with relative retention times and specific absorption maxima.

Peak No.	Pigments	Retention time (min)	Absorption maxima (nm)		
1	Chlorophyll *c_1_*+ *c_2 _*(Chl*c_1_*+ *c_2_*)	3.59	445	583	634
2	Unknown (RT 5.15)	5.15	423	446	476
3	Diadinoxanthin (Dd)	7.24	422	446	475
**4**	**Moraxanthin**	**8.07**	**421**	**444**	**472**
5	Diatoxanthin (Dt)	8.6	426	451	478
6	Zeaxanthin (Zea)	9.08		449	477
7	Chlorophyll *a* (Chl*a*)	14.25	432	617	665
8	b-carotene (b-car)	16.75		450	478

The new pigment was then isolated to determine its structure by spectroscopic (MS and NMR) means. A large-scale (10 L) culture of *C. cf. verruculosa* was grown, and harvested by continuous flow centrifugation into an algal pellet and supernatant. The algal pellet (4 g) was extracted exhaustively with MeOH, and the extract was subjected to repeated HPLC separation, yielding 1.1 mg of the pure carotenoid moraxanthin (**1**). When re-injected in the same HPLC conditions as for the chromatogram in Figure 2, the isolated compound **1** showed retention time and UV spectrum identical to that of peak 4. Compound **1** showed a [M + Na]^+^ pseudomolecolar ion peak in the ESI high-resolution mass spectrum at *m*/*z* 779.4879, in accordance with the formula C_48_H_68_NaO_7_. Compared to the C_40_ carotenoid skeleton, this formula contains eight additional carbon atoms. In addition, the ESI mass spectrum also contained a peak at *m*/*z* 663 (C_42_H_56_NaO_5_, [M + Na − C_6_H_12_O_2_]^+^), which can be accounted for by the in-source loss of hexanoic acid.

**Figure 4:**
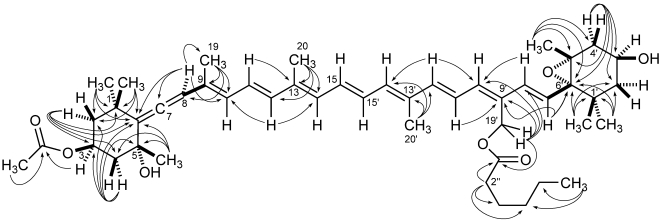
^1^H-^13^C long range couplings evidenced by the HMBC spectrum of moraxanthin (**1**).

Most of the information used for structure elucidation came from one- and two-dimensional NMR spectroscopy. The general features of the proton NMR spectrum (C_6_D_6_) resembled those of carotenoids, with several olefinic protons between δ 6 and δ 7, and 10 methyl singlets between δ 1.87 and 1.08. However, one of the ten methyl signals (δ 1.74) was part of an acetyl group, as shown by its correlation peak with the carbonyl carbon atom at δ 169.2 in the HMBC spectrum. Other notable features of the proton NMR spectrum were (i) the AB system at δ 5.13 and 5.07 (H_2_-19') of an oxymethylene group, (ii) two oxymethine protons at δ 5.69 (H-3) and δ 3.78 (H-3'), a methyl triplet at δ 0.78 (H_3_-6'') and a methylene triplet at δ 2.14 (H_3_-2''), indicative of an acyl chain and (iv) an olefinic proton singlet at δ 6.03 (H-8), which showed correlation peaks in the HMBC spectrum with two non-protonated carbon atoms at δ 202.1 (C-7) and 117.8 (C-6), and was therefore part of an allene system. The observed structural features were suggestive of a structure similar to vaucheriaxanthin (**2**), but a direct comparison of spectral data was hindered by the presence of the two additional acyl groups. A detailed analysis of the correlation peaks observed in the COSY, HSQC, and HMBC (Figure 4) spectra demonstrated that the planar structure of moraxanthin is indeed the same as that of vaucheriaxanthin, except for the presence of an acetyl group at position 3 and a hexanoyl group at position 19'.

In addition to all the expected geminal and vicinal couplings, the COSY spectrum revealed several proton-proton long-range couplings. Among them, the quite large W couplings of H-2α with H-4α (2.2 Hz) and of H-2'α with H-4'α (1.7 Hz) indicated the 1,3-diequatorial relationship of these two pairs of protons. Furthermore, the methyl protons on the polyene system showed weak correlation peaks with the olefinic protons, arising from the usual allylic ^4^*J*_HH_ couplings, but also from ^6^*J*_HH_ couplings (H_3_-19/H-12, H_3_-20/H-15', H_3_-20'/H-15) and even one remarkable ^8^*J*_HH_ coupling (H_3_-19/H-14). To the best of our knowledge, this is the first report of a ^8^*J*_HH_ coupling in a carotenoid. 

The *E* configuration of double bonds at positions 11, 15, 7', and 11' was evident from the large *trans* coupling constant values of the relevant protons (see Table 2). The *E* configuration of double bonds at positions 9, 13, and 13' and the *Z* configuration of the double bond at position 9' were determined from the ROESY spectrum, displaying correlation peaks of H_3_―19 with H-11, H_3_―20 with H-15, H_3_―20' with H-15', and H_2_―9' with H-11'.

**Table 2:** ^1^H (700 MHz) and ^13^C (175 MHz) NMR data of moraxanthin **1** in C_6_D_6_.

Pos.		δ_H _(*J* in Hz)	δ_C_, mult	Pos.		δ_H _(*J* in Hz)	δ_C_, mult
1		-	35.8, C	1'		-	35.2, C
2	α	2.05, ddd (12.4, 4.2, 2.2)	45.8, CH_2_	2'	α	1.48, ddd (12.8, 3.3, 1.7)	47.1, CH_2_
	β	1.39, dd (12.4, 11.5)			β	1.11, dd (12.8, 10.2)	
3		5 .69, dddd (11.5, 11.5, 4.2, 4.2)	67.7, CH	3'		3.78, ddddd (10.2, 8.6, 5.2, 4.3, 3.3)	63.7, CH
				3'-OH		0.57, d (4.3)	-
4	α	2.30, ddd (12.8, 4.2, 2.2)	45.5, CH_2_	4'	α	2.24, ddd (14.2, 5.2, 1.7)	41.0, CH_2_
	β	1.41, ddd (12.8, 11.5, 1.7)			β	1.48, dd (14.2, 8.6)	
5		-	72.0, C	5'		-	66.6, C
5-OH		0.77, d (1.7)	-			-	-
6		-	117.8, C	6'		-	69.9, C
7		-	202.1, C	7'		6.22, d (15.7)	125.9, CH
8		6.03, s	103.3, CH	8'		6.63, d (15.7)	134.9, CH
9		-	131.9, C	9'		-	132.4, C
10		6.20, d (11.4)	129.0, CH	10'		6.27, d (11.6)	136.3, CH
11		6.70, dd (15.0, 11.4)	125.3, CH	11'		6.95, dd (14.9, 11.6)	124.1, CH
12		6.46, d (15.0)	137.5, CH	12'		6.39, d (14.9)	141.0, CH
13		-	136.8, C	13'		-	136.4, C
14		6.28 , d (11.5)	133.0, CH	14'		6.27, d (11.5)	134.5, CH_2_
15		6.66, dd (14.3, 11.5)	131.1, CH	15'		6.60, dd (14.3, 11.5)	130.3, CH
16		1.43, s	29.0, CH_3_	16'		1.15, s	25.2, CH_3_
17		1.08, s	32.1, CH_3_	17'		1.14, s	29.4, CH_3_
18		1.17, s	30.9, CH_3_	18'		1.19, s	20.0, CH_3_
19		1.78, s	13.8, CH_3_	19'	a	5.07, d (12.3)	58.1, CH_2_
					b	5.13, d (12.3)	
20		1.87, s	12.7, CH_3_	20'		1.86, s	12.6, CH_3_
1''		-	172.8, C	Ac	CH_3_	1.74, s	20.8, CH_3_
2''		2.14, t (7.5)	34.2, CH_2_		CO	-	169.2, C
3''		1.55, quintet (7.5)	24.8, CH_2_				
4''		1.12, overlapped	31.3, CH_2_				
5''		1.14, overlapped	22.4, CH_2_				
6''		0.78, t (7.0)	13.8, CH_3_				

**Figure 5:**
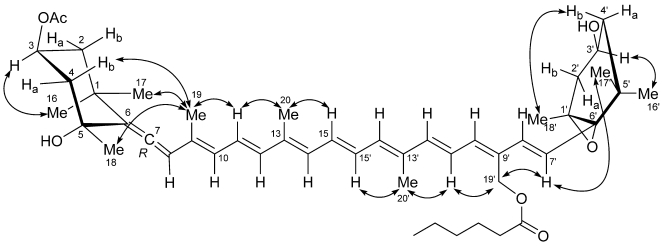
ROESY correlations used to determine the relative configuration of moraxanthin (**1**).

The ROESY spectrum also provided information on the relative configuration of the two terminal six-membered rings (Figure 5). The allene terminus is in the chair conformation, with the two W-coupled H-2α and H-4α protons in the equatorial orientation. The large coupling constants of H-3 with the axial H-2β and H-4β (Table 2) showed the former proton to be axial, and therefore on the α face of the ring; as a consequence, the OAc group at C-3 must be β. The ROESY correlation of the methyl protons H_3_-19 with H-2β and H-4β determined the axial chirality of the allene functionality as *R*. Finally, the ROESY correlation of H_3_-19 with H_3_-18 located C-18 on the β face of the ring, and therefore the OH group at C-5 on the α face.

As for the other terminal ring, the W coupling (1.7 Hz) of the pseudoequatorial H-2'α and H-4'α suggests a half-chair conformation of this ring. The *trans* relationship between the epoxide ring and the hydroxyl group was established from the ROESY correlation peaks of the two geminal methyl groups H_3_-16' and H_3_-17' with, respectively, H-3' and H-7' (Figure 5), showing that H-3' and H-7' are on opposite faces of the six-membered ring. This was confirmed by the prominent peak between the psudoaxial H-4β and H_3_-18 in the same spectrum. The relative configuration determined for moraxanthin matches that of vaucheriaxanthin (**2**), and it may be assumed that also the absolute configuration of moraxanthin is the same as in vaucheriaxanthin.

**Figure 6:**
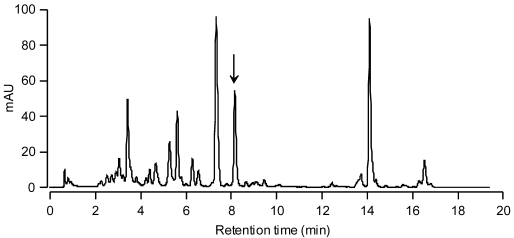
HPLC absorbance chromatogram of natural water sample collected at the fish-kill site of Torque Canal, Delaware on 28 August 2000 during a *C. cf. verruculosa* bloom. The arrow indicates the moraxanthin peak.

To investigate the utility of using moraxanthin as a marker for the toxic alga *C. cf. verruculosa*, natural bloom samples from the fish-kill site at Torque Canal, Delaware, collected on 28 August 2000 on Whatman GFF glass fiber filters and stored at −80 °C, were extracted in methanol and subjected to HPLC analysis. The HPLC chromatogram (Figure 6) definitely showed a peak for moraxanthin in the natural sample. The moraxanthin peak clearly separated from all the other pigment peaks having no overlap with other pigments. In addition, a peak with the same retention time and absorbance characteristics was present in the HPLC chromatogram from water samples collected in 2003-2007 at various sites in Delaware’s Inland Bays where *C. cf. verruculosa* blooms occurred (data not shown). This shows that the HPLC analysis may provide a simple and rapid tool for detecting harmful blooms of *C. cf. verruculosa*.

## 3. Experimental Section

### 3.1. General experimental procedures

ESI-MS experiments were performed on an Applied Biosystem API 2000 triple-quadrupole mass spectrometer. High Resolution ESI-MS spectra were performed on a Thermo Orbitrap XL mass spectrometer. All the mass spectra were recorded by infusion into the ESI source using MeOH as the solvent. CD spectra were recorded in MeOH solution on a Jasco J-710 spectrophotometer using a 1 cm cell. ^1^H and ^13^C NMR spectra were determined in C_6_D_6_ solution on a Varian UnityInova spectrometer at 700 and 175 MHz, respectively; chemical shifts were referenced to the residual solvent signal (δ_H_ 7.15, δ_C_ = 128.0). For an accurate measurement of the coupling constants, the one-dimensional ^1^H NMR spectra were transformed at 64K points (digital resolution: 0.09 Hz). Homonuclear ^1^H connectivities were determined by COSY experiments. Through-space ^1^H connectivities were evidenced using a ROESY experiment with a mixing time of 500 ms. The reverse multiple-quantum heteronuclear correlation (HMQC) spectra was optimized for an average ^1^*J*_CH_ of 142 Hz. The gradient-enhanced multiple-bond heteronuclear correlation (HMBC) experiment was optimized for a ^3^*J*_CH_ of 8.3 Hz. 

### 3.2. Plant material

Clonal cultures of *C. cf. verruculosa* were established by single cell pipette isolation from a natural bloom sample taken at the time of a fish-kill in Torque Canal, Delaware. Individually isolated cells were grown in DYV medium [43] using sea water adjusted to a salinity of 20 to match that of the sample water. Successful isolates grown in 96 well microtiter plates were stepped up in volume eventually becoming stabilized cultures maintained in 150 mL volumes in erlenmeyer culture flasks. All cultures were maintained at 22 °C, with a fluence rate of 50 µ mol. quanta m^−2^ s^−1^ of cool white fluorescent light and a 12:12 h (LD) cycle. For this study, culture CMS TAC1050 was used and is presently deposited in the Center for Marine Science Toxic Algal Collection housed at UNCW’s marine facility. This collection of harmful species is under the direction of Dr. Carmelo Tomas, Professor of Biology and Marine Biology at the CMS location who was also the isolator of the original culture. Large volume cultivation consisting of 10 L batches were grown in Bellco stirred cell system under conditions mentioned above. After a growth period of 1 month, the 10 L culture was harvested using a Sorvall RCB-2 refrigerated centrifuge equipped with a KSB (Kendro) continuous centrifuge head. A 4 g (wet weight) pellet was harvested, transferred to 15 mL cryovials and kept frozen at −80 °C prior to analyses for pigments.

### 3.3. Pigment analysis

The algal pellet (1 g) from cultures of *C. cf. verruculosa* was extracted with MeOH (3 mL), and the extract was filtered through Whatman GFF (0.45 µm). A portion of extract (500 μL) was added to 250 µL of ion-pairing solution (1M ammonium acetate), and after 5 minutes injected to the HPLC system. Assessment of the pigment composition was performed using a Hewlett-Packard HPLC 1100 Series system, equipped with a quaternary pump system and diode array detector. Pigments were separated on a temperature-controlled (20 °C) Hypersil MOS C8 reverse phase column (Sigma-Aldrich, 3 µm, 100 × 4.6 mm) according to the HPLC method of Vidussi *et al.* [45]. The mobile phases were MeOH (eluent A) and MeOH/0.5 N ammonium acetate (7:3) (eluent B). The elution gradient was kept constant at 1.0 mL/min for 20 min. The ratio of eluent B was gradually increased from 25 to 100%, and then returned to the initial proportion at the end of the elution. Chlorophylls and carotenoids were detected at 440 nm and identified by a diode array detector (λ = 350-750 nm, 1.2 nm spectral resolution). Standards of all the known pigments were provided by International Agency for ^14^C Determination (VKI Water Quality Institute) and calibration was performed according to Mantoura and Repeta [46]. 

### 3.4. Analysis of algal bloom

Samples from blooms occurring in the Delaware Inland Bays were collected and returned to the laboratory or shipped by overnight courier to UNCW CMS Laboratory. Upon arrival, the sample was processed immediately. Pigment samples were taken as natural samples filtered on Whatman GFF filters and frozen immediately in liquid nitrogen and stored in a −80°C freezer until extraction and pigment analyses could be performed as described above. Species contents of the sample water were determined by direct observations using a Nikon Diaphot inverted microscope. Observations of the phytoplankton included species identification, at least to genus level of live cells, cell density estimates using standard inverted microscope techniques and extraction for lipid soluble toxins in chloroform. Preserved samples (Lugol’s solution) were carefully mixed, placed into a 10 or 50 mL settling chamber and allowed to settle for 24 hours prior to observation. The dominant species were identified, enumerated and used to define the phytoplankton composition.

### 3.5. Extraction and isolation of moraxanthin (**1**)

The algal pellet (4 g) was extracted once with MeOH (40 mL). The extract was dried to give a dark green oil (104 mg) which was subjected to reversed-phase HPLC separation on a Varian Prostar 210 apparatus equipped with an Varian 325 UV detector [column: RP-18, 10 *μ*m, 250 ´ 10 mm; eluent: MeOH/H_2_O (9:1), flow 5 mL/min, UV detector set at 430 nm] to give partially purified moraxanthin (3.4 mg). Further reversed phase HPLC [column: RP-18, 3 *μ*m, 250 ´ 4.6 mm; eluent: MeOH/H_2_O (8:2), flow 1 mL/min, UV detector set at 430 nm] gave pure moraxanthin **1** (1.1 mg), whose identity was confirmed by HPLC analysis as described in Section 4.3. 

### 3.6. Moraxanthin (**1**)

Dark yellow oil; CD (MeOH; *c* 3.06 10^-6^ M): Δε_471_ +9.9, Δε_446_ +11.9, Δε_422_ +9.0; UV (EtOH): *λ*_max_ nm (*ε*): 421 (89000), 444 (129000), 472 (118000); ESI MS *m*/*z* 779 [M + Na]^+^; HRESIMS *m*/*z* 779.4879 [M + Na]^+^ (calcd. for C_48_H_68_NaO_7_, 779.4857). For ^1^H and ^13^C NMR spectroscopic data, see Table 2.

## 4. Conclusions

The newly proposed toxic species *C. cf. verruculosa* contains a new species-specific pigment, moraxanthin (**1**), whose structure was established as (3S,5*R*,7*R*,3'*S*,5'*R*,6'*S*)-3-acetoxy-5',6'-epoxy-19'-(hexanoyloxy)-6,7-didehydro-5,6,5',6'-tetrahydro-β,β-carotene-5,3'-diol, *i.e.*, 3-*O*-acetyl-19'-*O*-hexanoylvaucheriaxanthin. Two esterified forms of vaucheriaxanthin have been described, namely the 3-*O*-acetyl-19'-*O*-octanoate and the 3-*O*-acetyl-19'-*O*-decanoate derivatives [47]. However, none of them contains the hexanoyl residue present in moraxanthin, which therefore can be easily distinguished from these known compounds on the basis of the HPLC retention time. 

New harmful species have been identified and the taxonomyof other species has been revised [48]. It is usually accepted that the routineidentification of phytoplankton for monitoring studies in estuariesand coastal waters requires additional methods other than traditionalmicroscopy, which can underestimate some taxonomic groups containingfragile or poorly differentiated small cells. In conjunction withmicroscopy, pigment separation using HPLC has becomea more widely applied method for estimating and characterizing phytoplanktonbiomass and community structure [6,7,8].

However, algal pigments usually show complex overlapping patterns with different taxa, offering only a few unambiguous markers. In our case, *C. cf. verruculosa* may be readily identified in natural samples by means of HPLC chromatograms due to the distinct peak corresponding to the species-specific pigment moraxanthin described here.

## Appendix

Supplementary File 1 Supplementary file (PDF, 424 KB) Description: Supplementary Information
