# Supplementary material for: The New Carotenoid Pigment Moraxanthin Is Associated with Toxic Microalgae
Source: Mar Drugs. 2011 Feb 10;9(2):242–55. doi: 10.3390/md9020242 (PMC3093255; doi:10.3390/md9020242)
Supplement: Supplementary File 1 — Supplementary file (PDF, 424 KB) Description: Supplementary Information [file marinedrugs-09-242-s001.pdf]

The New Carotenoid Pigment Moraxanthin is Associated with a Toxic Microalgae

Olga Mangoni,[1] Concetta Imperatore,[2] Carmelo R. Tomas,[3] Valeria Costantino,[2] Vincenzo Saggiomo,[4] Alfonso Mangoni[2,]*

[1] Dipartimento delle Scienze Biologiche, Università di Napoli Federico II, Via Mezzocannone 8, 80134, Napoli, Italy

[2] Dipartimento di Chimica delle Sostanze Naturali, Università di Napoli Federico II, via D. Montesano 49, 80131 Napoli, Italy

[3] Center for Marine Science, University of North Carolina at Wilmington, 5600 Marvin K. Moss Lane, Wilmington, NC 28409, USA

[4] Stazione Zoologica "A. Dohrn", Villa Comunale I, 80121 Napoli, Italy

ELECTRONIC SUPPLEMENTARY INFORMATION

Table of contents

ESI mass spectrum of moraxanthin (**1**)....................S2
CD spectrum of moraxanthin (**1**)....................S2
^{1}H-NMR spectrum of moraxanthin (**1**)....................S3
COSY spectrum of moraxanthin (**1**)....................S4
COSY spectrum of moraxanthin (**1**) – high-field region....................S5
COSY spectrum of moraxanthin (**1**) – low-field region....................S6
HSQC spectrum of moraxanthin (**1**) – high-field region....................S7
HSQC spectrum of moraxanthin (**1**) – mid-field region....................S8
HSQC spectrum of moraxanthin (**1**) – low-field region....................S9
HMBC spectrum of moraxanthin (**1**)....................S10
HMBC spectrum of moraxanthin (**1**) – high-field region....................S11
HMBC spectrum of moraxanthin (**1**) – low-field region....................S12
ROESY spectrum of moraxanthin (**1**)....................S13
ROESY spectrum of moraxanthin (**1**) – high-field region....................S14

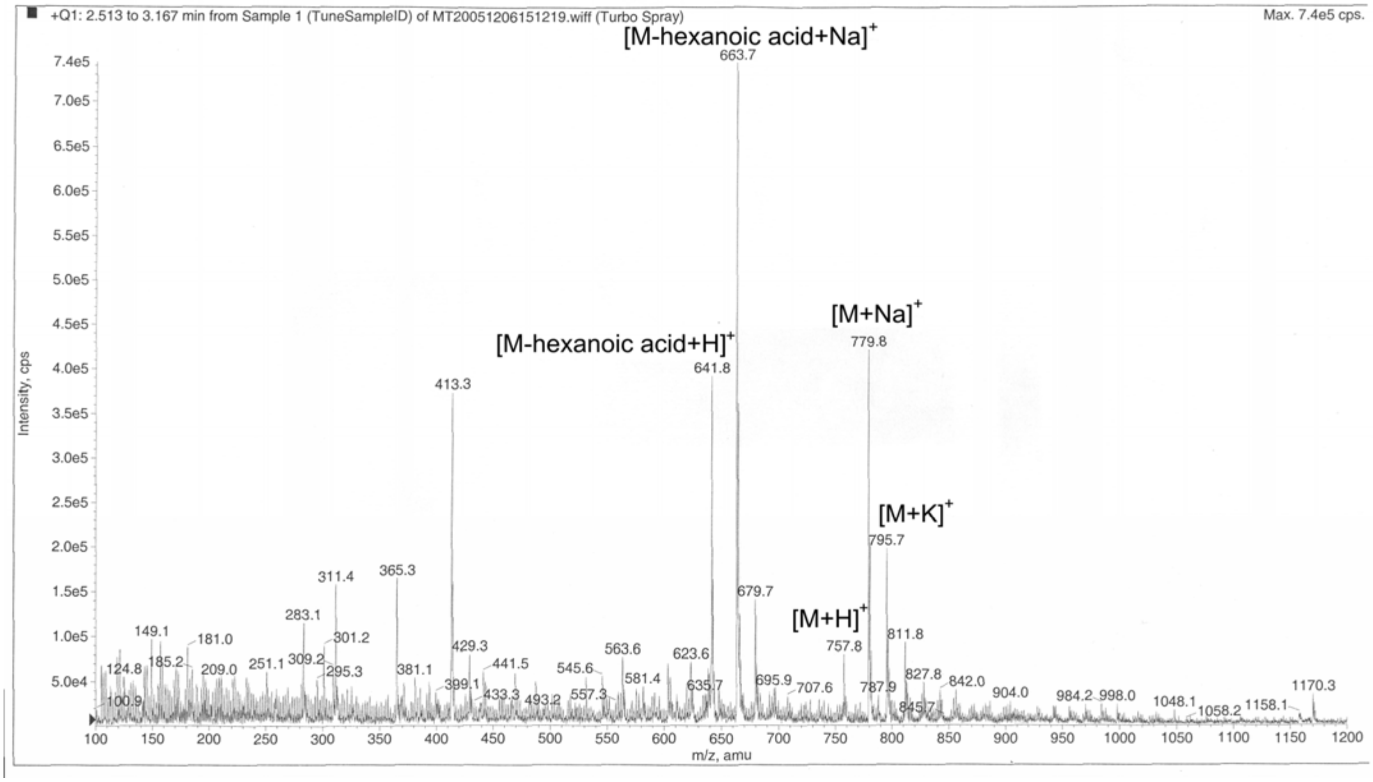

Moraxanthin (**1**). ESI MS spectrum

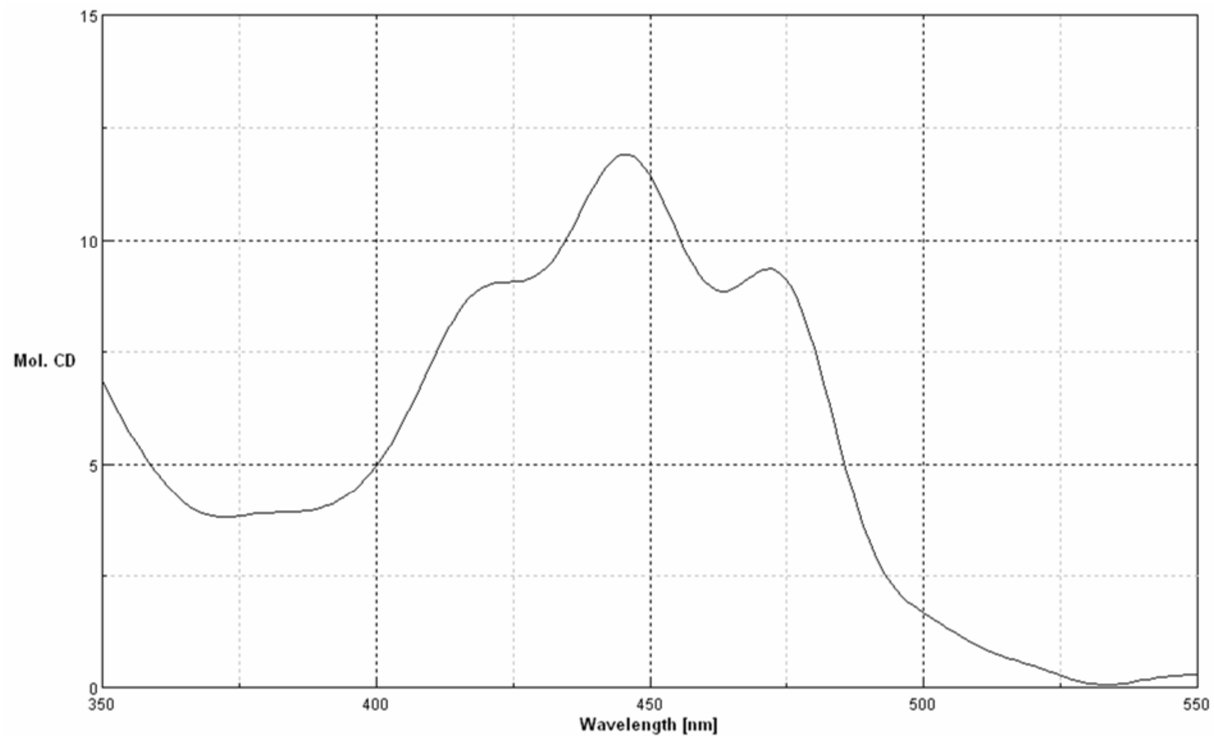

Moraxanthin (**1**). CD spectrum (MeOH)

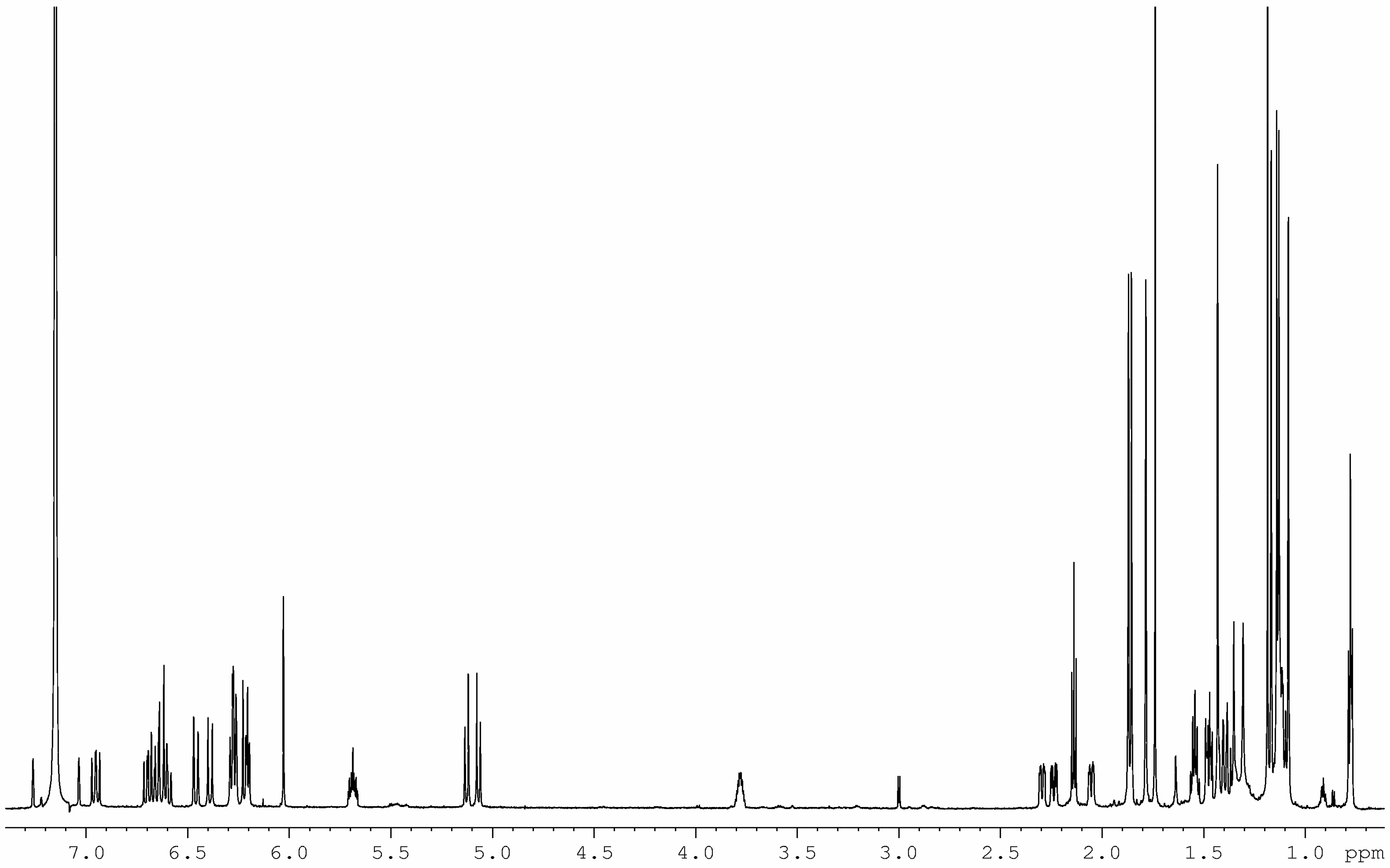

Moraxanthin (**1**) – ^{1}H NMR spectrum (C_6C_6, 700 MHz)

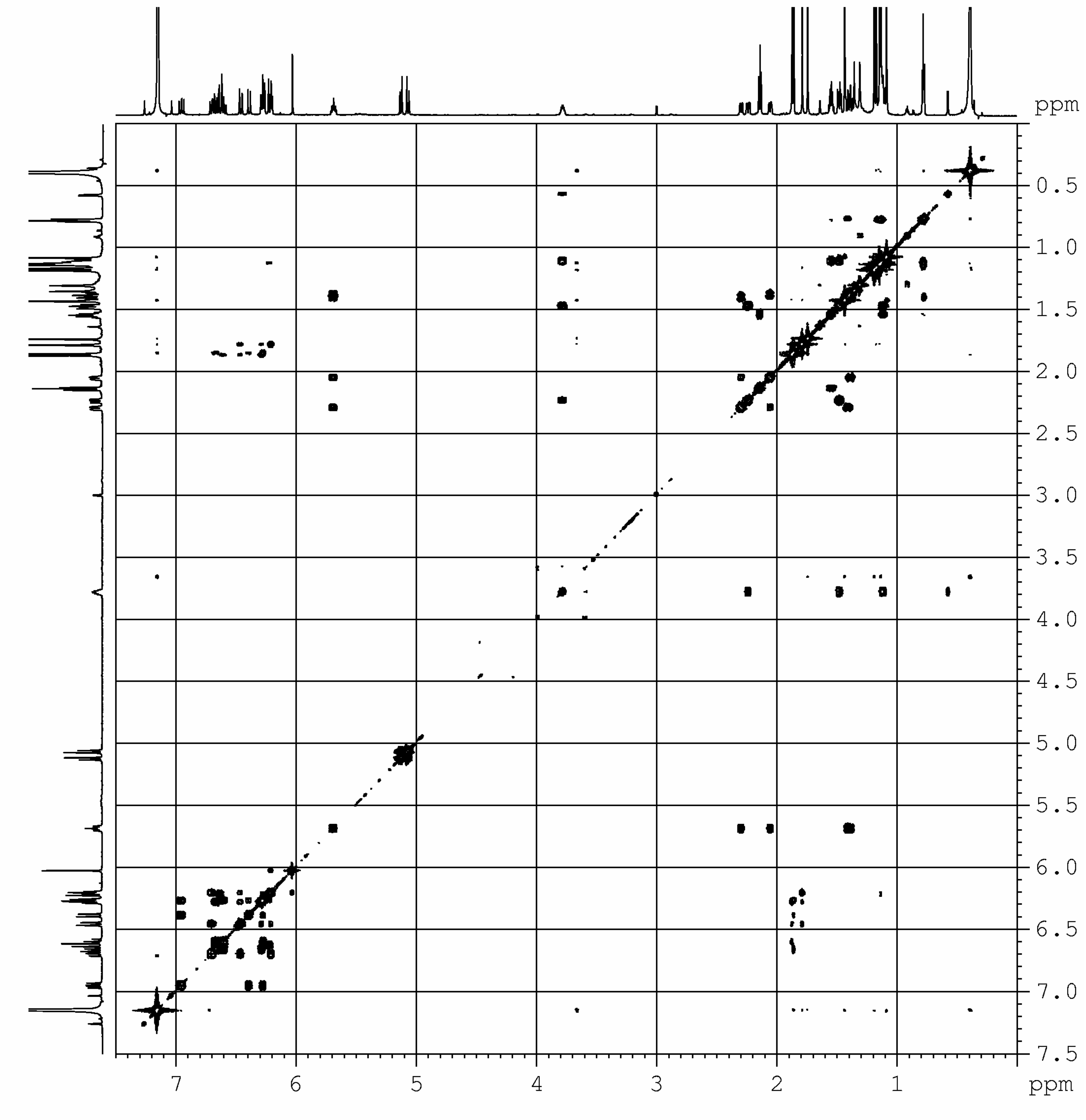

Moraxanthin (**1**) – COSY spectrum (C_6C_6, 700 MHz)

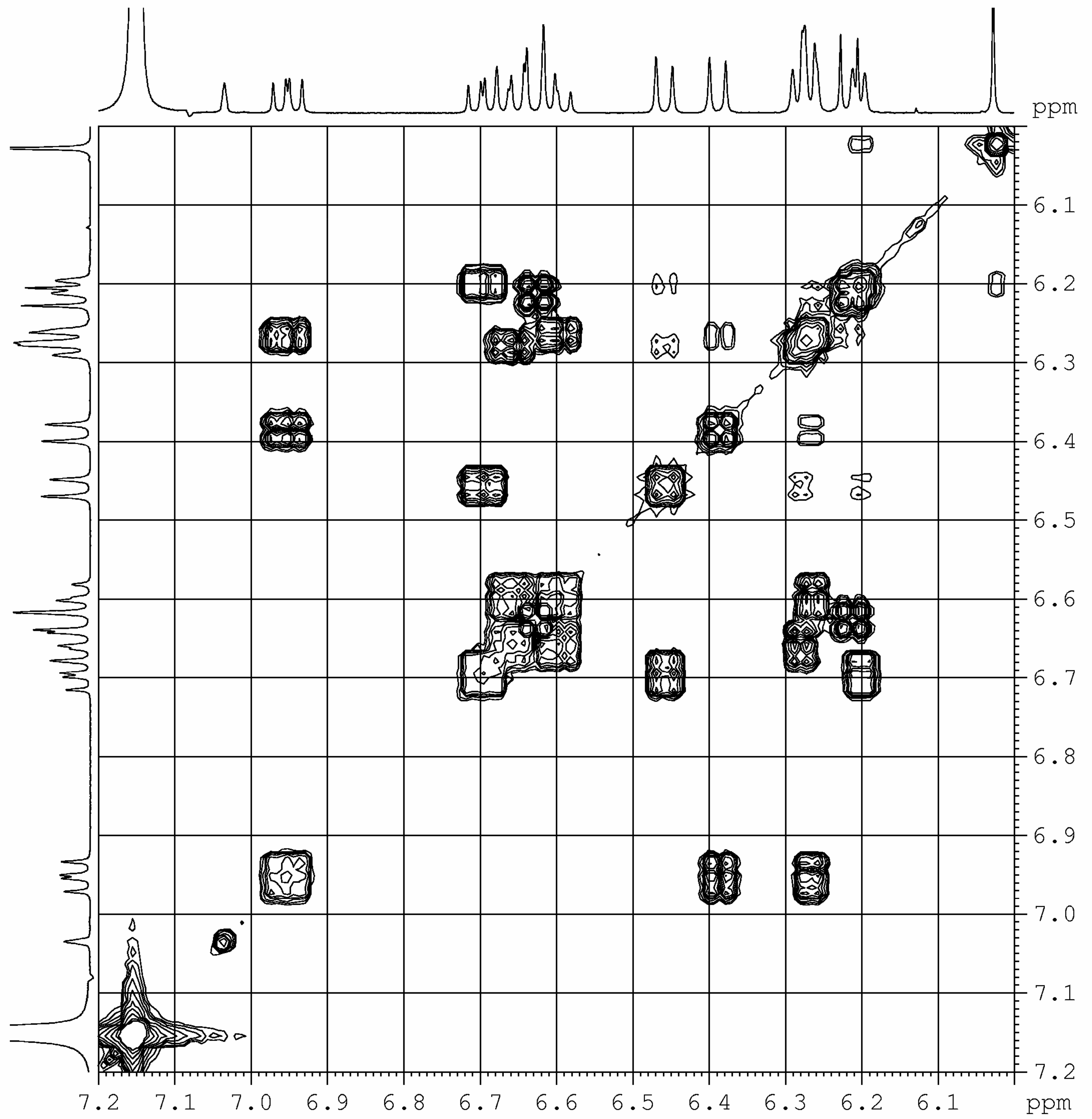

Moraxanthin (**1**) – COSY spectrum (C_6D_6, 700 MHz) – low-field region

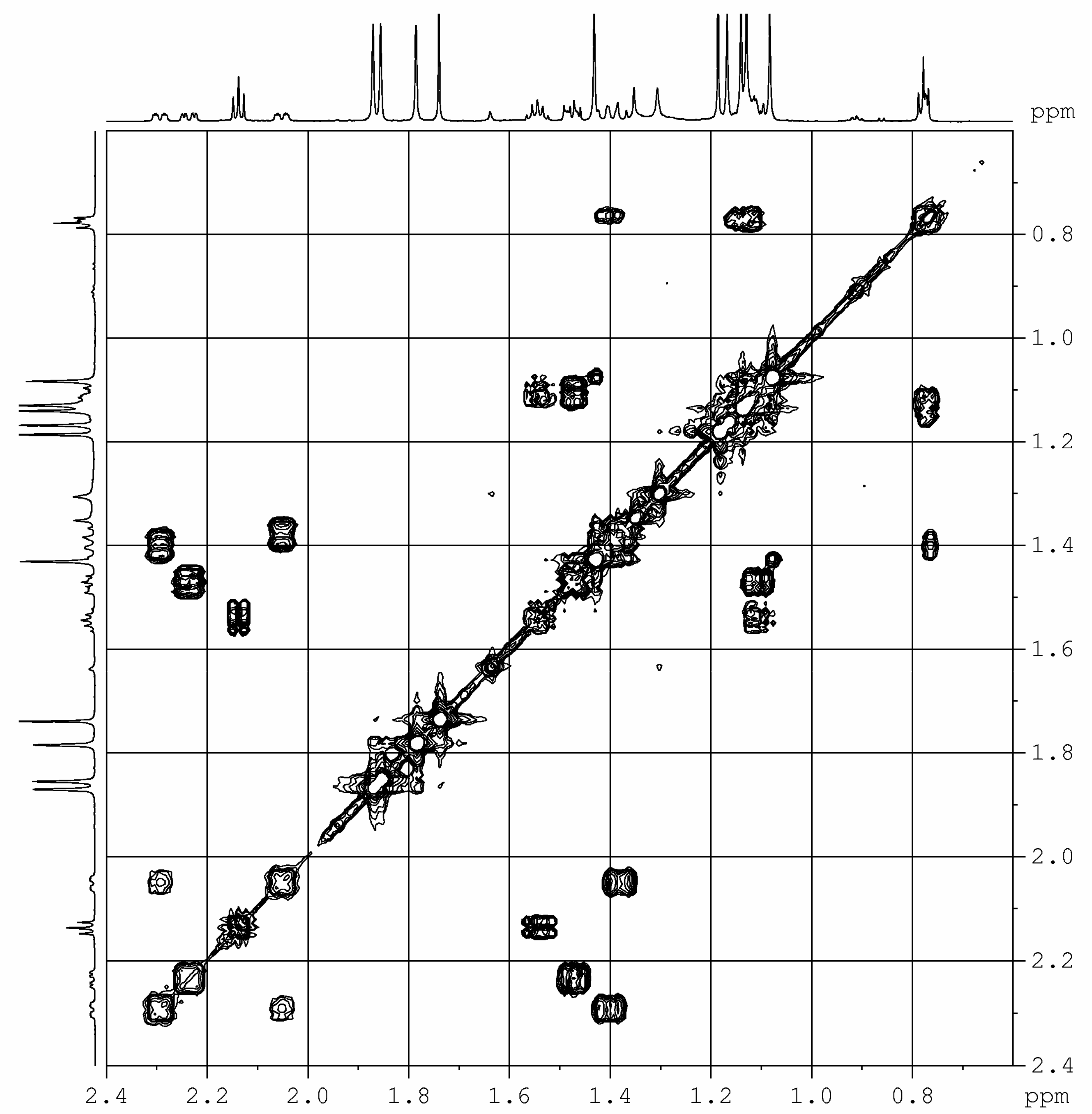

Moraxanthin (**1**) – COSY spectrum (C_6D_6, 700 MHz) – high-field region

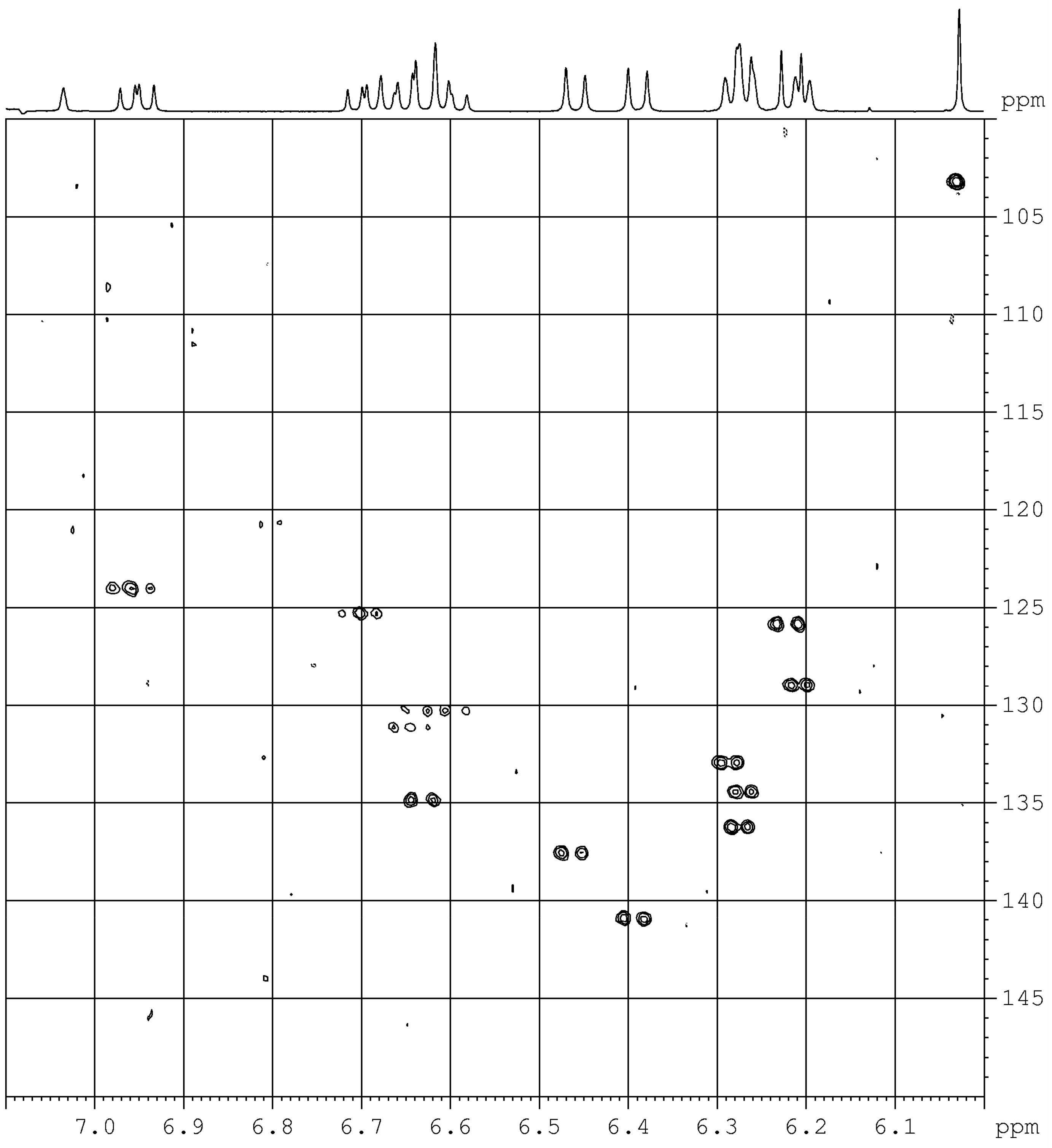

Moraxanthin (**1**) – HSQC spectrum (C_6D_6, 700 MHz) – low-field region

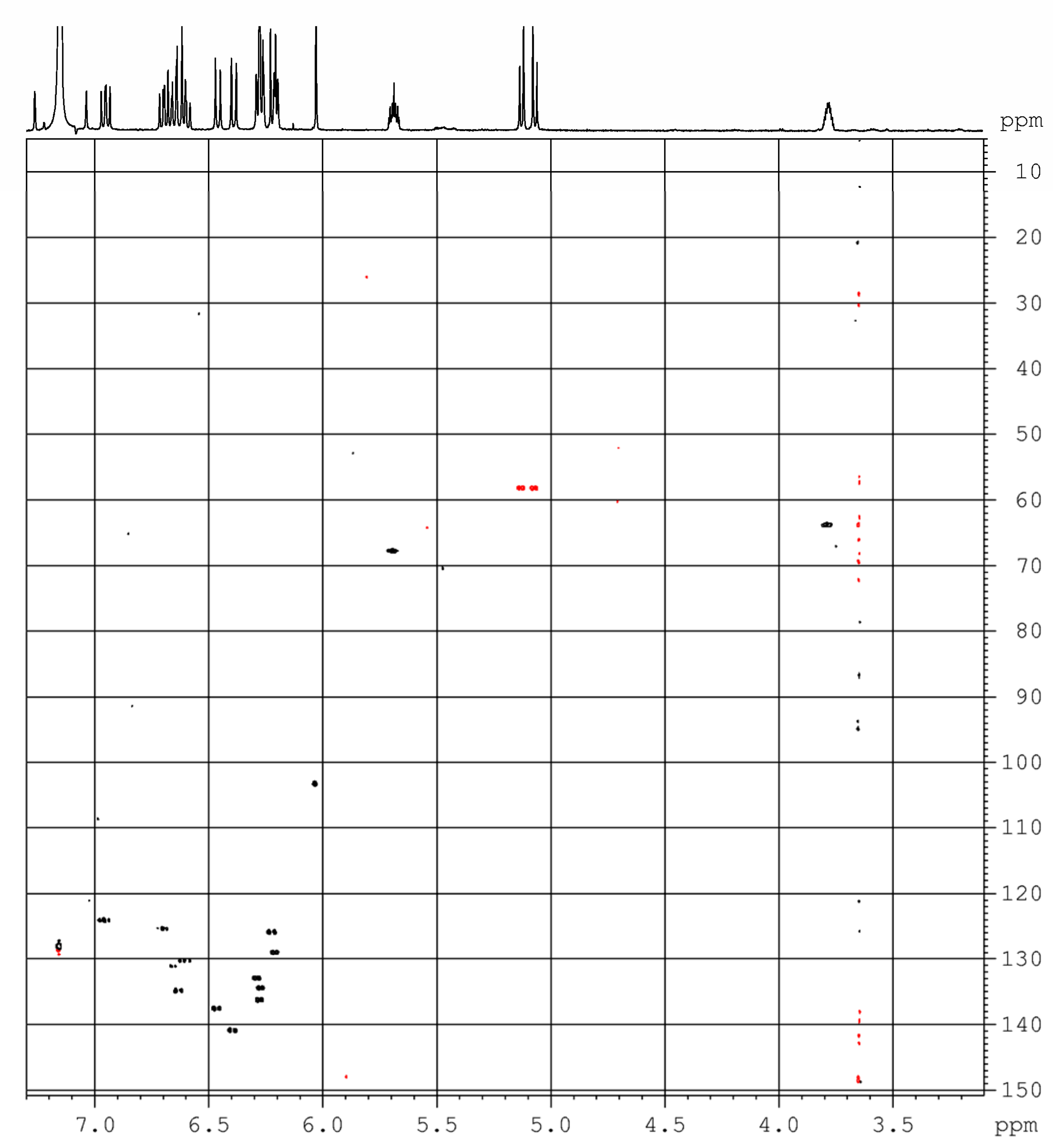

Moraxanthin (**1**) – HSQC spectrum (C_6D_6, 700 MHz) – middle-field region

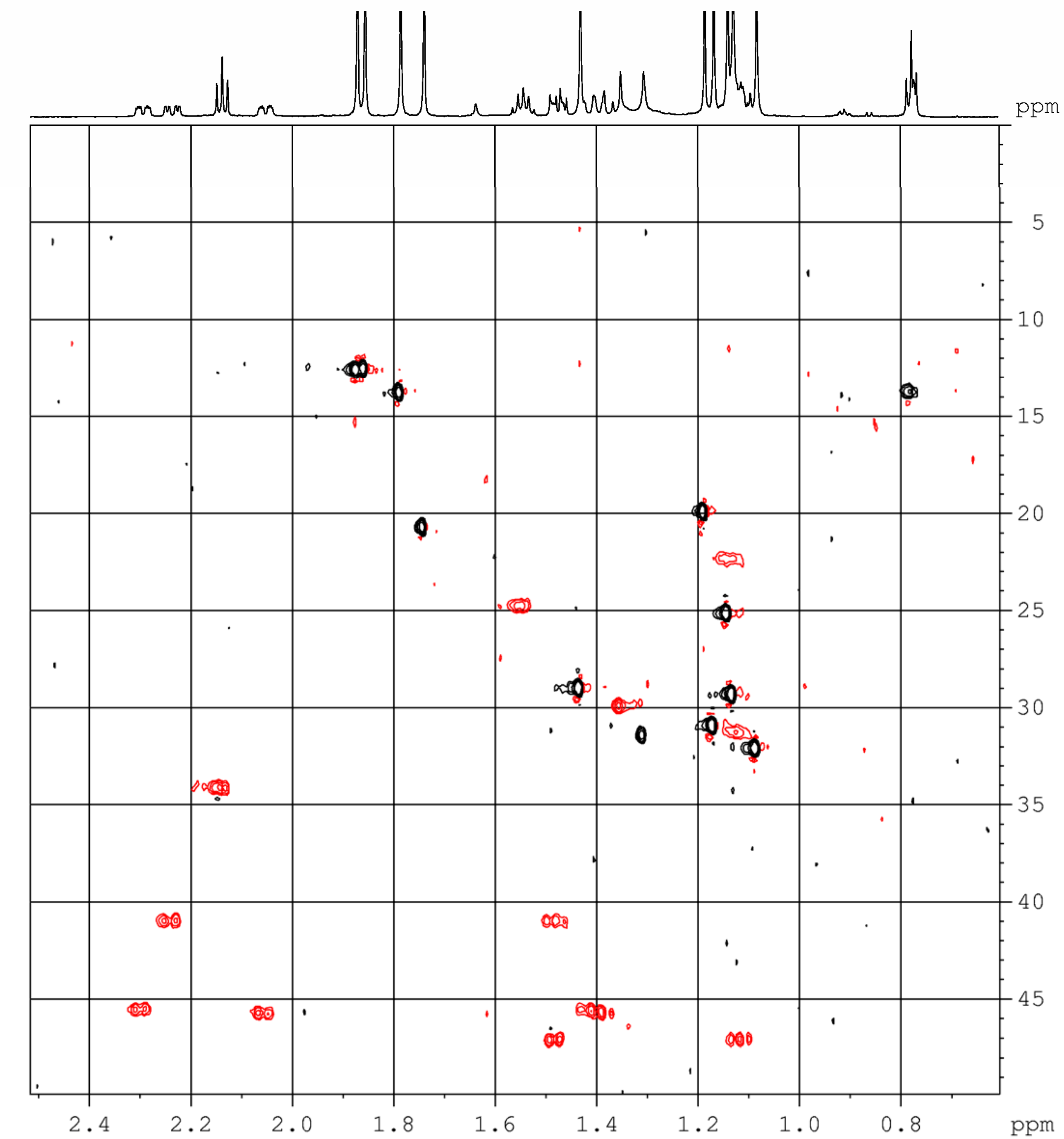

Moraxanthin (**1**) – HSQC spectrum (C_6D_6, 700 MHz) – high-field region

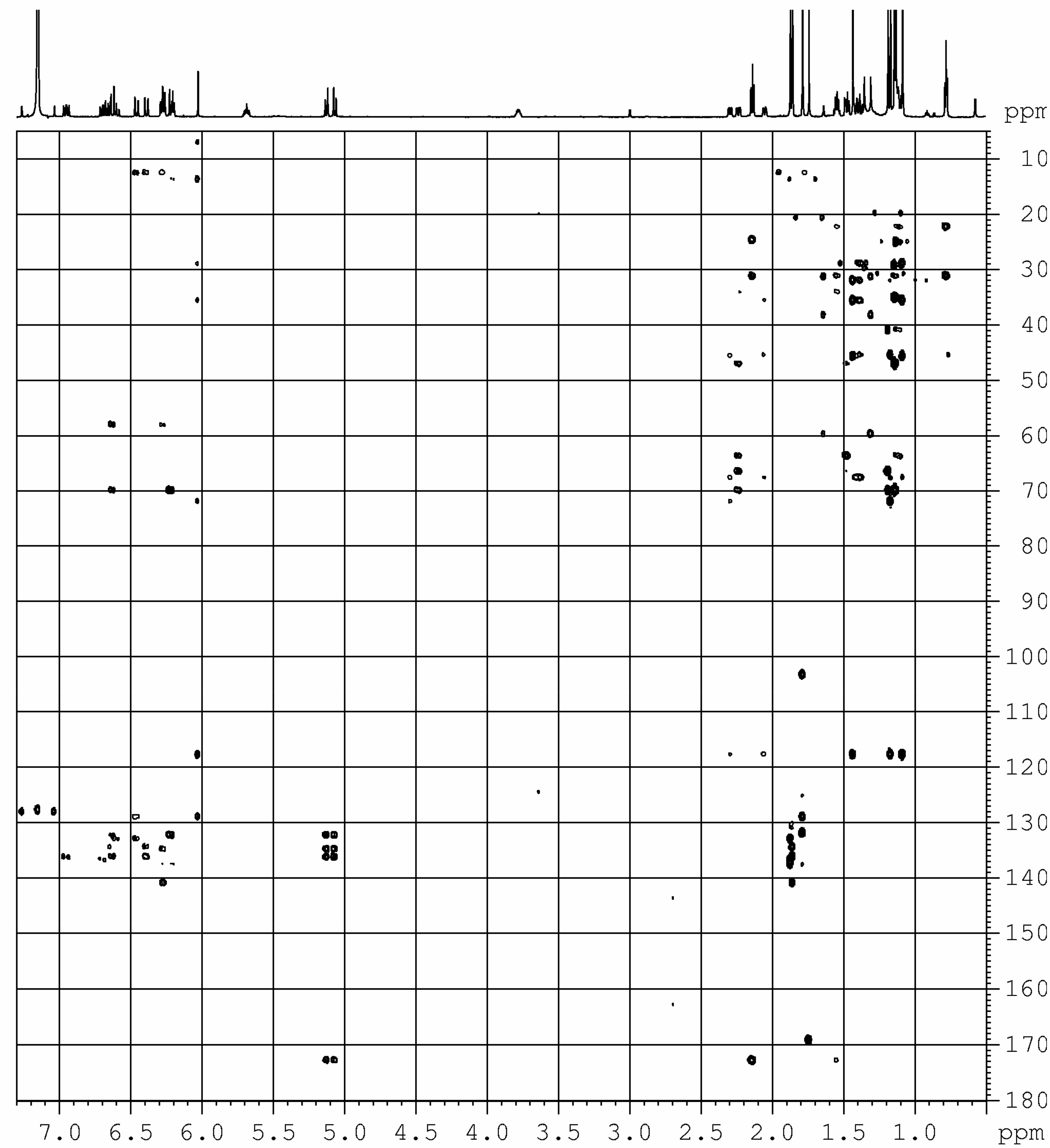

Moraxanthin (**1**) – HMBC spectrum (C_6D_6, 700 MHz

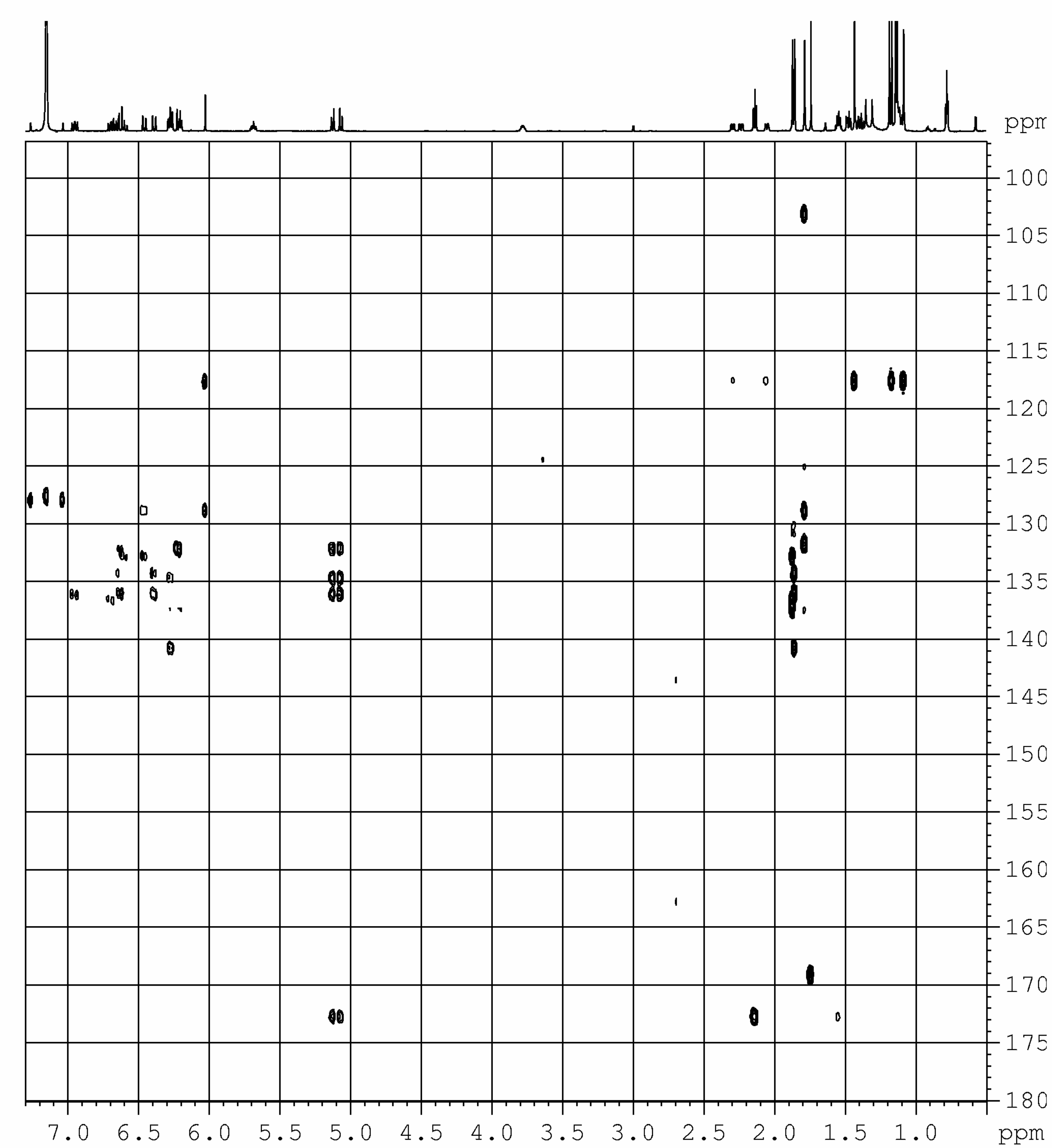

Moraxanthin (**1**) – HMBC spectrum (C_6D_6, 700 MHz) – low-field region

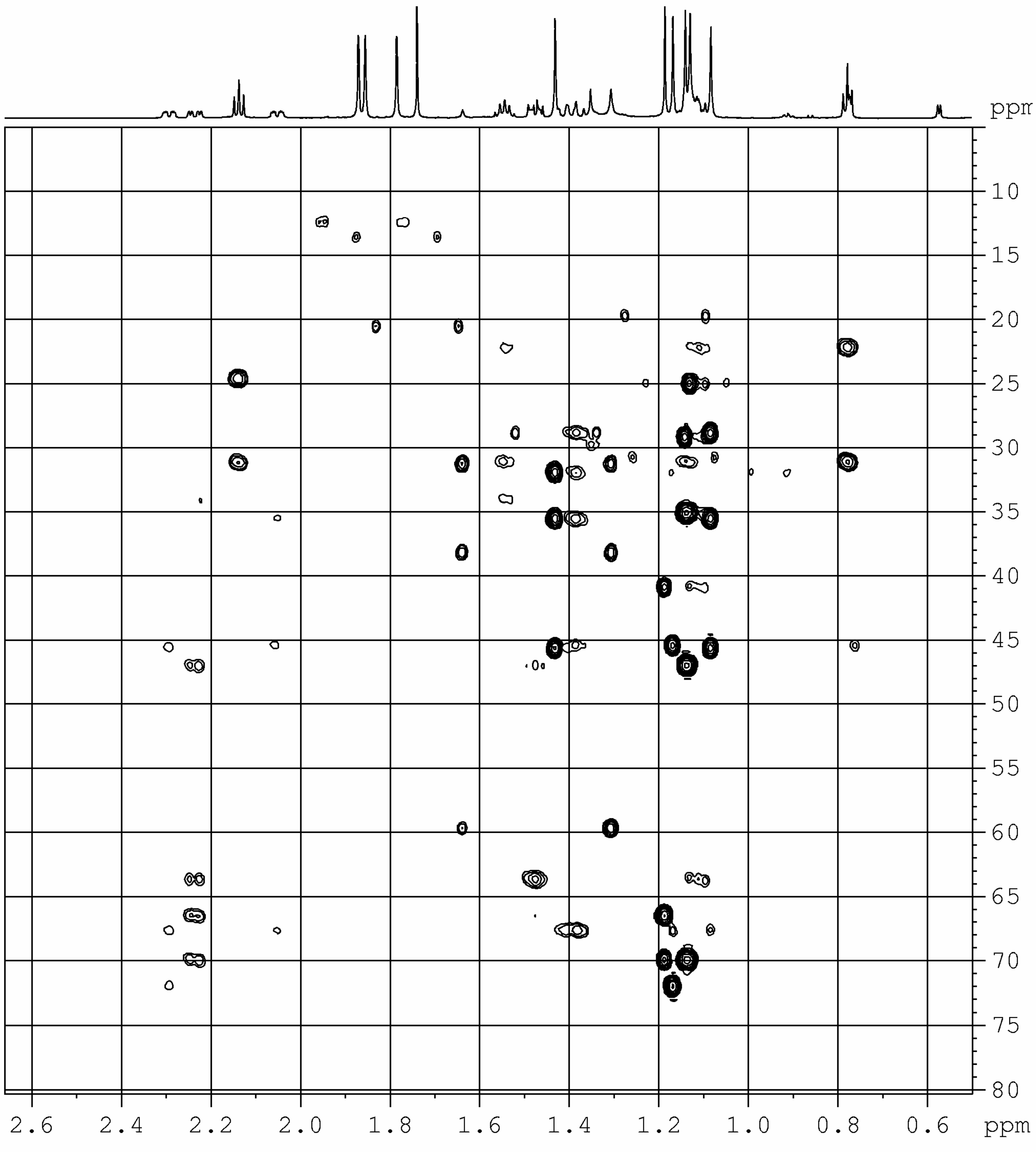

Moraxanthin (**1**) – HMBC spectrum (C_6D_6, 700 MHz) – high-field region

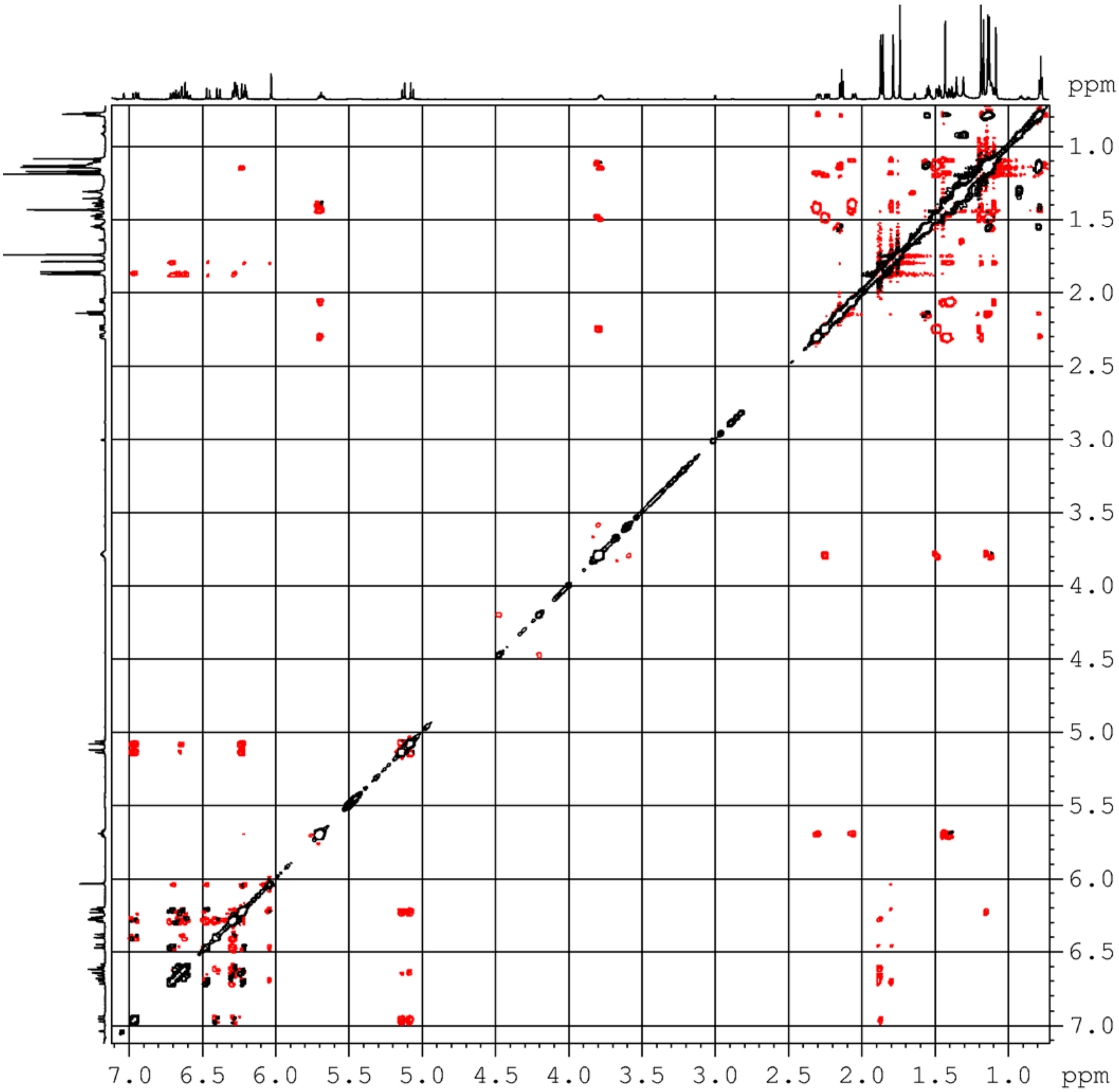

Moraxanthin (**1**) – ROESY spectrum (C_6D_6, 700 MHz

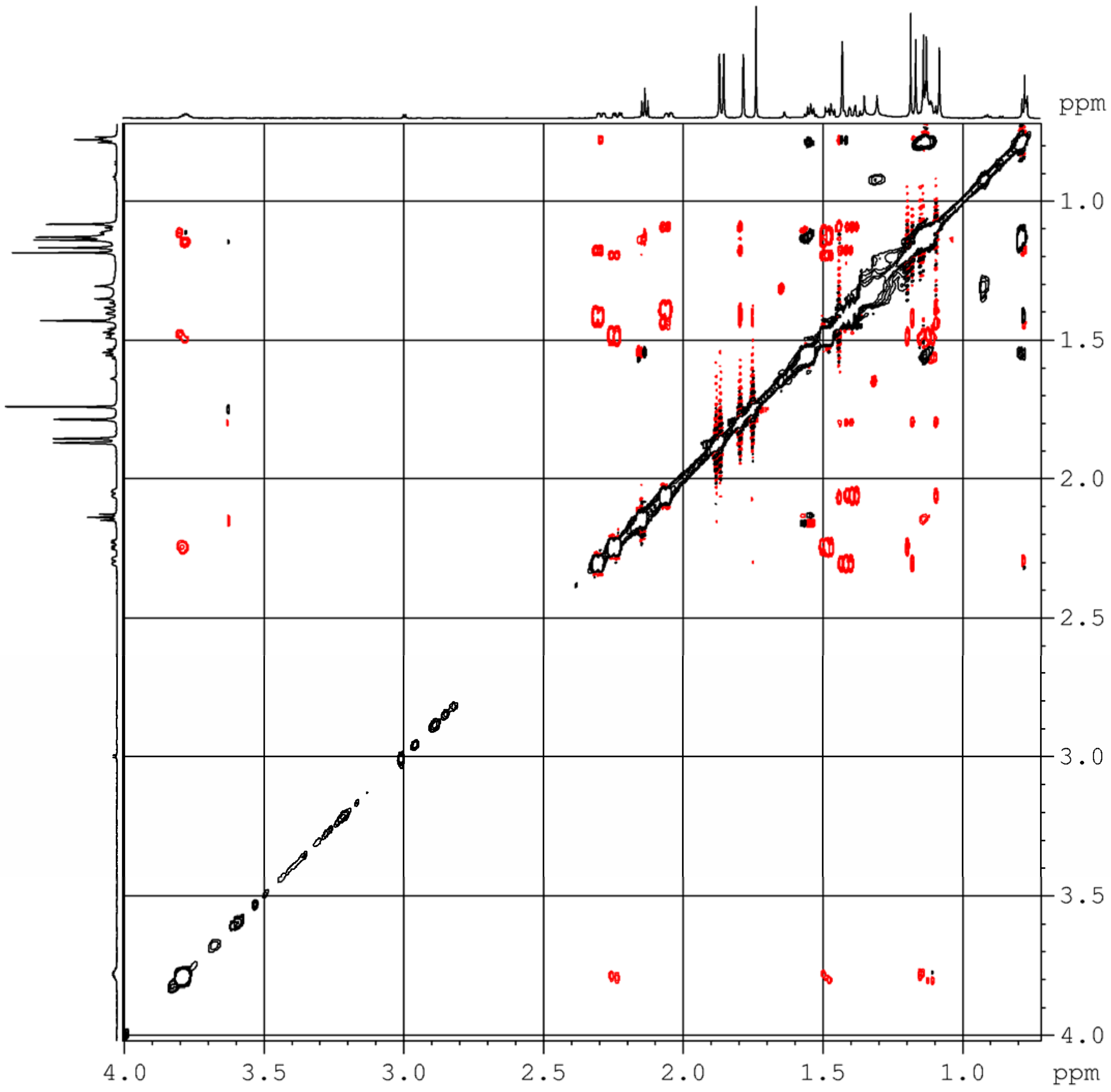

Moraxanthin (**1**) – ROESY spectrum (C_6D_6, 700 MHz) – high-field region
